# Charge-Regulated Electrochemistry in Self-Standing Carbon Nanotube–Nanocellulose Hybrid Electrodes

**DOI:** 10.1021/acs.langmuir.6c01676

**Published:** 2026-05-12

**Authors:** Laura Ferrer Pascual, Golnoosh Akhlamadi, Khadijeh Nekoueian, Maedeh Akhoundian, Mengqi Sun, Kristoffer Meinander, Henrikki Liimatainen, Tomi Laurila

**Affiliations:** † Department of Electrical Engineering and Automation, 174277 Aalto University, Maarintie 8, Espoo 02150, Finland; ‡ Department of Bioproducts and Biosystems, School of Chemical Engineering, 174277 Aalto University, PO Box 16300, Aalto 00076, Finland; § Fiber and Particle Engineering, 6370 University of Oulu, P.O. Box 4300, Oulu 90014, Finland; ∥ Department of Chemistry and Materials Science, Aalto University, Kemistintie 1, Espoo 02150, Finland

## Abstract

Self-standing electroactive thin films enable direct interrogation of intrinsic structure-composition-property relationships without contributions from external current collectors. Here, we report a bioderived hybrid thin film composed of single-walled carbon nanotubes (SWCNTs) and TEMPO-oxidized cellulose nanofibers (TOCNFs) that functions simultaneously as a conductive scaffold and an electrochemically active sensing interface. The hybrid architecture integrates the hydrophobic, conductive SWCNTs with the hydrophilic, carboxyl-functionalized TOCNF network to form a mechanically robust, porous film with composition-tunable interfacial charge and electron transfer properties. By systematically varying the SWCNT:TOCNF ratio and electrolyte conditions, we elucidate how hybrid composition governs charge distribution, redox probe accessibility, and electron transfer kinetics. The optimized self-standing films exhibit reproducible, composition-dependent electrochemical behavior and enable electrostatic discrimination between oppositely charged redox species. These findings provide mechanistic insight into charge-regulated electrochemistry in carbon nanotube–nanocellulose hybrids and establish composition-controlled charge regulation as a design principle for self-supporting electroactive thin films.

## Graphical abstract

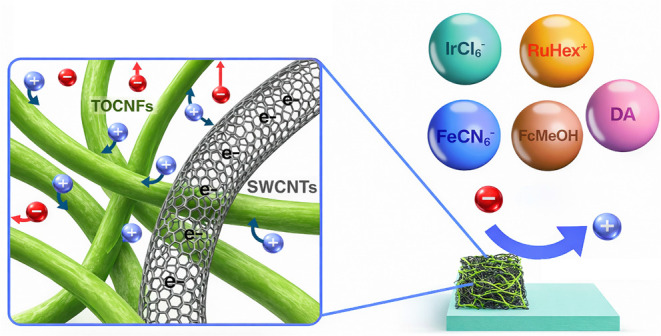

## Introduction

Hybrid nanomaterials have emerged as architected thin-film systems in which electronic transport, mechanical integrity, and interfacial chemistry can be simultaneously engineered. Among these, carbon nanotube (CNT) networks integrated with nanocellulose matrices provide a versatile platform for constructing porous, mechanically robust films with tunable interfacial properties. Nanocellulose contributes hydrophilicity, chemical functionality, film-forming capability,^1–4^ and resistance to fouling^5,6^ while CNTs provide high electrical conductivity, large surface area, and mechanical resilience.^7–10^ When assembled into percolated porous networks, these components form lightweight, flexible, and aqueous-processable thin films whose electroactive behavior arises from the interplay between conductive pathways and chemically functional, charge-bearing nanocellulose domains. Such hybrid architecture offers a sustainable route toward electroactive materials in which structure, charge distribution, and surface chemistry are intrinsically linked.

CNT-nanocellulose composited have been exploited in energy-storage applications, including self-standing films for batteries and supercapacitors.^11–15^ In parallel, CNT-nanocellulose layers have been investigated in electrochemical sensing platforms. However, in sensing configurations these hybrids are typically deposited onto preexisting conductive substrates such as glassy carbon or tetrahedral amorphous carbon electrodes,^16–20^ where the underlying current collector contributes to charge transport and interfacial behavior. Conversely, self-standing CNT-nanocellulose films developed for energy storage have not been systematically examined as independent electrodes for electrochemical sensing. As a result, the intrinsic electrochemical response of a fully self-supporting CNT-nanocellulose thin film acting simultaneously as a mechanical scaffold, conductive network, and sensing interface remains unexplored. This distinction is critical because removing the current collector fundamentally alters how charge percolation, electrostatic screening, and analyte accessibility are coupled within the porous network. When the hybrid film itself is the sole conductive and interfacial material, electrochemical behavior is governed entirely by the internal architecture and composition of the composite. This configuration enables direct interrogation of intrinsic structure-composition-property relationships that are otherwise convoluted by substrate-induced contributions. To the best of our knowledge, this work represents the first demonstration of fully self-supporting CNT-nanocellulose thin films functioning as standalone electrochemical sensing electrodes, operating without any external current collector.

Here, we report a self-standing hybrid thin film composed of single-walled carbon nanotubes (SWCNTs) and TEMPO-oxidized cellulose nanofibers (TOCNFs) that operates as an independent electrode for electrochemical sensing without external conductive support. By systematically varying the SWCNT:TOCNF ratio and electrolyte conditions, we demonstrate that electrochemical behavior is governed not solely by bulk conductivity, but by composition-dependent charge regulation within the nanostructured porous matrix. Using outer-sphere and inner-sphere redox probes as interfacial reporters, we show that background charge distribution and nanoscale organization collectively regulate ion accessibility and electron-transfer kinetics. In this architecture, conductive CNT pathways and charged nanocellulose domains coexist throughout the thin film, directly linking electron transport to local electrostatic environments. Consequently, interfacial kinetics can be rationally tuned through hybrid composition.

Although the present study focuses on TEMPO-oxidized cellulose nanofibers (TOCNFs), which impart a net negative background charge to the hybrid network, the electrochemical behavior of CNT-nanocellulose hybrids is not inherently restricted to a single charge state. The surface chemistry of the nanocellulose component can be systematically modified to produce neutral or positively charged nanofibers, enabling deliberate tuning of the background electrostatic landscape while preserving the conductive scaffold. This chemical modularity establishes composition-controlled charge regulation as a general materials design principle for self-supporting electroactive thin films, providing a framework for engineering porous hybrid electrodes in which nanoscale architecture and interfacial electrostatics are intentionally integrated.

## Materials and Methods

### Materials

Bleached cellulose pulp was used as the cellulose source for preparing TEMPO-oxidized cellulose nanofibers (TOCNF) following our previously reported method.^25^ 2,2,6,6-Tetramethylpiperidine-1-oxyl radical (TEMPO, ≥98%), sodium bromide (NaBr, ≥99%), sodium hypochlorite solution (NaClO, 14% active chlorine), polyethylenimine (PEI) solution (a branched polymer, average Mn ∼ 60,000 by gel permeation chromatography, average Mw ∼ 750,000 by light scattering, 50 wt % in H_2_O), hexaammineruthenium­(III) chloride, tetracycline hydrochloride, potassium ferricyanide, ferrocene methanol, hexachloroiridate, dopamine hydrochloride, and phosphate buffer saline (PBS, pH 7.4) were obtained from Sigma-Aldrich. Long-length single-wall carbon nanotubes (SWCNT; diameter ≈ 1 nm, length up to 30 μm, purity > 92%) were purchased from Nanografi.

### Preparation of SWCNT:TOCNF Hybrid Films

Hybrid dispersions of SWCNT and TOCNFs were produced following the method described in our previous work.^21^ In brief, predispersed SWCNT and TOCNF were sonicated under controlled conditions and then centrifuged to obtain a homogeneous and stable dispersion. The supernatant was subsequently collected. The optimized hybrid compositions (1:1 and 2:1 SWCNT:TOCNF, 30 min sonication) were used for all electrochemical measurements in this study. The electrodes were prepared by drop-casting a 0.1 wt % PEI solution onto a glass slide and allowing it to stand for 10 min. The coated slide was then rinsed three times with deionized water (DIW) and dried under a nitrogen stream. Subsequently, the hybrid solution was spin-coated onto the PEI-modified glass slide at 4000 rpm for 1 min, followed by drying at 70 °C for 30 min.

### Electrochemical Measurements

Electrochemical measurements were carried out using both conventional cyclic voltammetry (CV) and differential pulse voltammetry (DPV). A Gamry Reference potentiostat was used with a three-electrode setup containing an Ag/AgCl reference electrode and a platinum wire as a counter electrode. The solutions were purged with N_2_ gas for 15 min before the measurements. Newly prepared electrodes (0.07 cm^2^ geometrical area) were used for each electrochemical measurement. All the measurements were carried out at room temperature in the Faraday cage.

All experiments are performed with freshly prepared electrodes, and the peak current values are obtained with baseline correction and presented as the mean and standard deviation of three electrodes (*N* = 3).

The pseudocapacitance is calculated from the electrolyte cyclic voltammograms by subtracting the difference between the anodic (*i_a_*) and cathodic (*i_c_*) currents at a specific potential and scan rate (*v*):^22^

$$C_{pseudo}=\frac{i_{a}-i_{c}}{2v}$$

### Atomic Force Microscopy

Atomic force microscopy (AFM) images were acquired in Peak Force Quantitative Nanomechanical Mapping (PFQNM) mode using the AFM instrument (Bruker Dimension Icon, USA). To successfully image our coated surfaces, a silicon nitride cantilever (Scanasyst-fluid+) was used, featuring a nominal spring constant of 0.7 N/m and a resonance frequency of 150 kHz. All imaging experiments were conducted in a buffer medium (PBS) and at room temperature (20–23 °C). Conductive AFM (cAFM) measurement was performed using the AFM Jupiter XR (Oxford Instruments Asylum Research) in the air. The cantilever ASYELEC-01-R2 with a radius of 25 nm and the frequency of 75 kHz was applied for this measurement. The AFM images were processed using Gwyddion software (version 2.69).

### Raman Spectroscopy

Raman spectra were recorded using a Renishaw inVia^TM^ Qontor Raman microscope equipped with a 532 nm (green) excitation laser. The hybrid films were prepared by drop-casting the hybrid dispersions onto PEI-coated glass substrates and drying at 70 °C for 15 min prior to measurement.

### X-ray Photoelectron Spectroscopy

X-ray photoelectron spectroscopy (XPS) was performed using a Kratos AXIS Ultra DLD X-ray photoelectron spectrometer with a monochromated Al_Kα_ X-ray source (1486.7 eV) run at 100 W. Photoelectrons were collected at a 90° takeoff angle under ultrahigh vacuum conditions, with a base pressure typically below 1 × 10^–9^ Torr. Survey spectra were collected with a pass energy of 80 eV and a step size of 1.0 eV, while high-resolution spectra were collected with a pass energy of 20 eV and a step size of 0.1 eV. All spectra were charge-corrected relative to the position of the C–O carbon in cellulose, located at 286.5 eV, which corresponds to a binding energy of 284.8 eV for aliphatic C–C carbon. Peak fitting was done with the CasaXPS software, using Gaussian–Lorentzian peaks of equal full-width-at-half-maximum and standard tabulated binding energies for each individual high-resolution region.^23^

## Results

### Structural Characterization

Figure 1A shows the morphology of the 1:1 and 2:1 SWCNT:TOCNF hybrids. The 1:1 hybrid exhibited a fibrillar and relatively open network, suggesting a less compact structure that may facilitate the diffusion of larger molecules into the network. In contrast, the 2:1 hybrid has a denser, more entangled appearance dominated by SWCNT bundles, which may hinder molecular accessibility and diffusion into the hybrid network. This issue will be discussed in more detail later on. It is difficult to distinguish between TOCNFs and SWCNTs based solely on conventional AFM images, as both species exhibit similar dimensions. However, since SWCNTs are expected to have much higher conductivity than TOCNFs, spreading-resistance AFM can be used to differentiate their respective contributions. The corresponding measurements are shown in Figure 1B and C. As evident from the images, some SWCNTs have aggregated into larger bundles that exhibit significantly higher currents (as indicated with blue lines) compared to the surrounding matrix. Some assemblies of TOCNFs with lower conductivity can also be seen (as indicated with pink lines). The SWCNT bundles form a conductive network whose overall conductivity is expected to depend on the number and stability of interbundle contacts.

**Figure 1:**
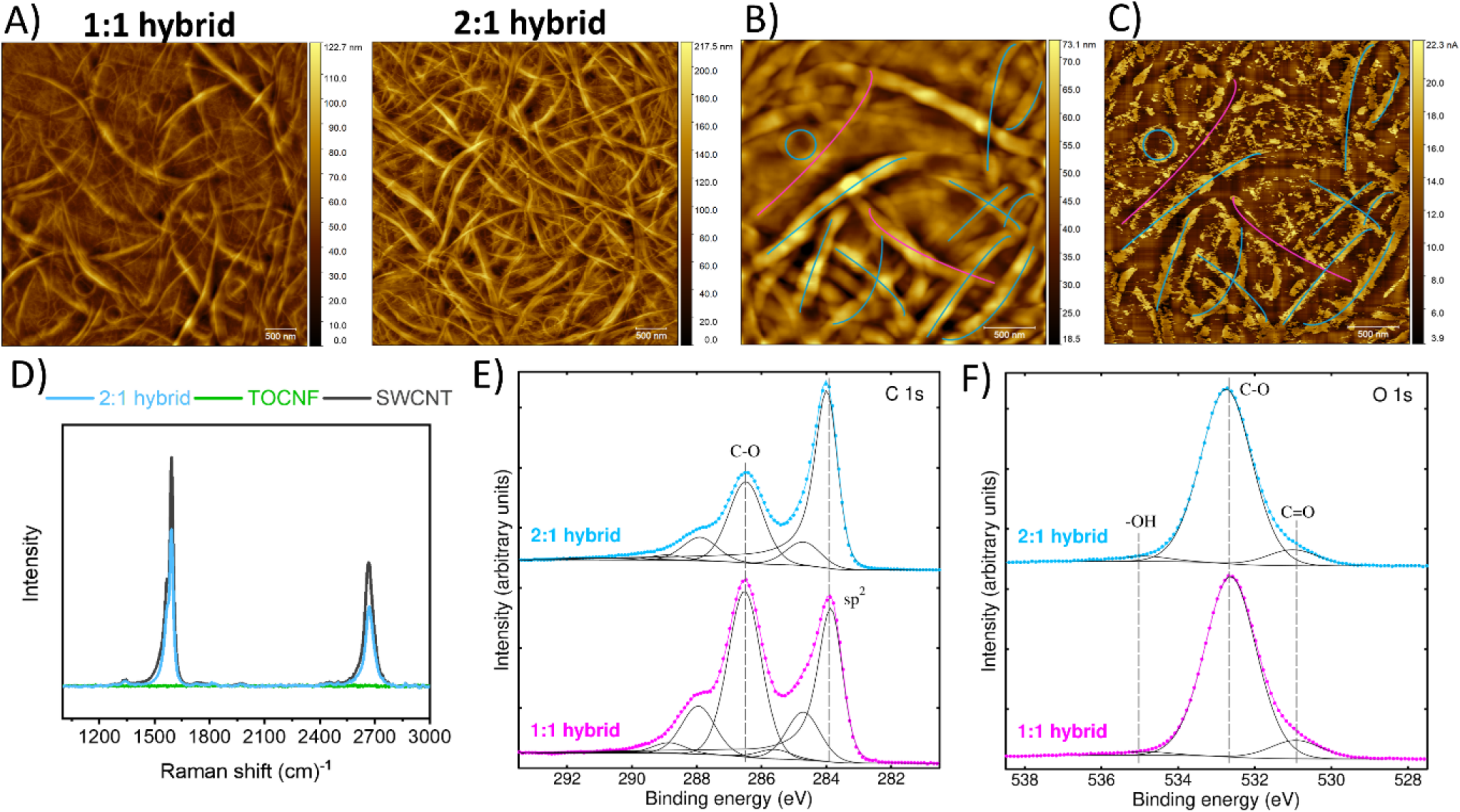
(A) AFM images of 1:1 and 2:1 hybrid. The roughness was of 9.31 nm (1:1 hybrid) and 23.01 nm (2:1 hybrid). cAFM images of (B) height and (C) corresponding current map of 2:1 hybrid. High conductivity areas are marked with blue lines and low conductivity areas are marked with pink lines. (D) Raman spectra of pristine SWCNTs, TOCNFs, and the 2:1 hybrid. (E–F) High-resolution XPS spectra of C 1s and O 1s.

Sheet resistance measurements verified the strong dependence of conductivity on the CNT/TOCNF ratio. The 1:1 hybrid shows a sheet resistance of 20.5 ± 5.8 kΩ sq^–1^, which is significantly higher than that of the 2:1 hybrid (1.80 ± 0.6 kΩ sq^–1^), Table 1. The much lower sheet resistance of the 2:1 hybrid in comparison to 1:1 is expected to arise from its higher SWCNT loading that promotes the formation of SWCNT bundles that constitute the conductive network as shown in Figure 1B and C.

**Table 1:** Structural, Chemical, and Electrical Properties of SWCNT:TOCNF Hybrids

Property	1:1 hybrid	2:1 hybrid
SWCNT (wt %)	0.125	0.250
TOCNF (wt %)	0.125	0.125
C 1s (sp^2^, %)	36.4	57.7
C 1s (oxygenated species, %)	51.0	35.6
O/C ratio	0.67	0.34
Charge density (mmol g^–1^)	0.7	0.7^a^
Sheet resistance (kΩ sq^–1^)	20.5 ± 5.8	1.80 ± 0.6
SWCNT/:TOCNF interaction	Physical (noncovalent)	Physical (noncovalent)

a The surface charge density was measured only for the TOCNF and does not correspond to the actual surface charge of the hybrid.

Raman spectroscopy (Figure 1D) provides direct evidence that the interaction between TOCNF and SWCNT is primarily physical. Both pristine SWCNT and the 2:1 hybrid display the characteristic D- and G-bands at approximately 1350 cm^–1^ and 1590 cm^–1^, respectively, with a distinct 2D band near 2680 cm^–1^. The negligible shift in band positions and the nearly unchanged I_D_/I_G_ ratio indicate that no significant chemical modification or covalent bonding took place during hybrid formation. Maintaining the intrinsic electronic structure of the SWCNT ensures that the conductive pathways remain intact which is a key factor for subsequent electrochemical performance.

The XPS results (Table 1, Figure 1E–F) further support this conclusion. Both hybrids are composed mainly of carbon and oxygen, with small amounts of sodium and chlorine. Trace amounts of silicon, calcium, magnesium and potassium from the glass substrate, as well as nitrogen from the PEI could also be detected. The relative intensity of the oxygenated carbon species (C–O, C=O, and O–C=O) is higher in the 1:1 hybrid reflecting the larger fraction of cellulose-derived oxygen functionalities, while the 2:1 hybrid shows a higher contribution from sp^2^ carbon and a reduced O/C ratio, consistent with its SWCNT-rich content. The surface charge density of the TOCNF is 0.7 mmol g^–1^, providing a negative background in the hybrid network. The O 1s spectra for both hybrids were dominated by the C–O peak near 532.8 eV, typical for cellulose-containing samples.

Overall, the AFM, Raman, XPS, and conductivity analyses demonstrate that the SWCNT:TOCNF hybrids form physically interconnected networks in which the cellulose nanofibers serve as a negatively charged scaffold for the SWCNTs, without altering their intrinsic electronic properties. The results further indicate that the electrical performance of the hybrids scales with the amount of SWCNTs incorporated into the material. However, as discussed in detail in,^21^ this relationship is not strictly linear but involves more complex dependencies.

### Electrochemical Characterization

The hybrid electrodes with different SWCNT:TOCNF ratios were compared in terms of their pseudocapacitance using different electrolyte strengths (Table 2). The pseudocapacitance of the hybrid originates from oxygen-containing surface groups on the SWCNTs, which explains the larger pseudocapacitive response in the samples with a higher nanotube loading (2:1 hybrid). Reducing the KCl concentration from 1.0 to 0.1 M increases the Debye length and alters the structure of the electrical double layer. The hydrophilic CNFs and partly oxidized SWCNT surfaces enable aqueous infiltration under both electrolyte conditions, ensuring that the near-surface region remains readily wetted. Although the lower ionic strength reduces counterion availability and slows ion transport into deeper pore regions, cyclic voltammetry primarily probes regions of the material that are already efficiently accessed by the electrolyte. Consequently, only modest changes in pseudocapacitance are observed when the electrolyte concentration is reduced. The slight variation in the magnitude and direction of the electrolyte-concentration dependence between the different hybrid ratios can be attributed to differences in fixed negative charge within the network and the balance between ionic accessibility and exposed SWCNT surface area. These effects together result in a relatively stable pseudocapacitive response over the tested electrolyte range.

**Table 2:** Values of Pseudocapacitance (μF/cm^2^) of the SWCNT:TOCNFs Hybrids in 0.1 and 1 M KCl

	Pseudocapacitance (μF/cm^2^)	
	0.1 M KCl	1 M KCl
1:1 hybrid	16.2 ± 0.6	18.9 ± 1.2
2:1 hybrid	51.2 ± 3.2	50.6 ± 2.2

The developed hybrid nanocellulose-SWCNT sensor was characterized with cationic, anionic and neutral outer sphere redox probes as well as inner sphere redox probe, such as dopamine.

### Outer-Sphere Redox Probe Characterization

Outer-sphere redox (OSR) probes retain a strong solvation shell, preventing direct chemical or electrocatalytic interaction with the electrode surface. As a result, electron transfer proceeds without specific adsorption or surface complexation. Nevertheless, their electrochemical response remains sensitive to the electrostatic environment at the electrode–electrolyte interface, in addition to the electronic structure of the electrode (e.g., surface density of states). Surface functional group charge and supporting electrolyte ionic strength can differentially affect cationic and anionic OSR probes, thereby offering insights into the surface charge properties and electron-transfer behavior of hybrid electrodes. Importantly, these effects primarily arise from mass transport effects influenced by interfacial electrostatics rather than changes in intrinsic electron-transfer kinetics.

To probe these effects, four OSR couples of different charge and structure (Ru­(NH_3_)_6_^3+/2+^, IrCl_6_^2–/3–^, Fe­(CN)_6_^3–/4–^, and FcMeOH^0/+^) were employed as model redox systems.

### Cationic Probe Ru­(NH_3_)_6_^3+/2+^

The redox couple Ru­(NH_3_)_6_^3+/2+^ was selected as a representative cationic outer-sphere probe to evaluate the electron-transfer behavior of the hybrid electrodes. Because Ru­(NH_3_)_6_^3+/2+^ undergoes electron transfer without requiring specific adsorption or surface complexation, it is commonly used as a benchmark to assess the intrinsic conductivity and interfacial kinetics of new electrode materials.

However, in our case the cyclic voltammograms of Ru­(NH_3_)_6_^3+/2+^ displayed abnormally shaped and broadened peaks, together with additional oxidation peaks not characteristic of this well-behaved OSR probe (Figure 2A, Table 3). These additional oxidation peaks (located ∼ 0.4 and 0.5 V) could be attributed to redox-active surface functionalities present within the SWCNT:TOCNF hybrid material. We observed that when the potential was scanned to values more negative than approximately −0.5 V, these surface-associated oxidation peaks appeared reproducibly, suggesting that reductive activation of surface oxygenated groups occurs at these potentials. When the potential window was limited to a narrower range, avoiding negative potentials beyond −0.5 V, the additional oxidation peaks were not observed, confirming that their appearance is triggered by prior reductive activation of the electrode surface (Figure S2). The additional peaks also appeared in the blank electrolyte solution (Figure 2B), confirming that they originate from the electrode surface rather than from Ru­(NH_3_)_6_^3+/2+^. Upon repeated potential cycling, these peaks gradually diminished or disappeared within ∼10 cycles, indicating that the corresponding redox species are not covalently bound but likely physisorbed or weakly associated with the hybrid matrix. After pausing the cycling for an extended period, the peaks tended to reappear, suggesting that not all impurities are removed upon cycling and some stay within the network, thus gradually return back to the electrode surface when cycling is paused.

**Figure 2:**
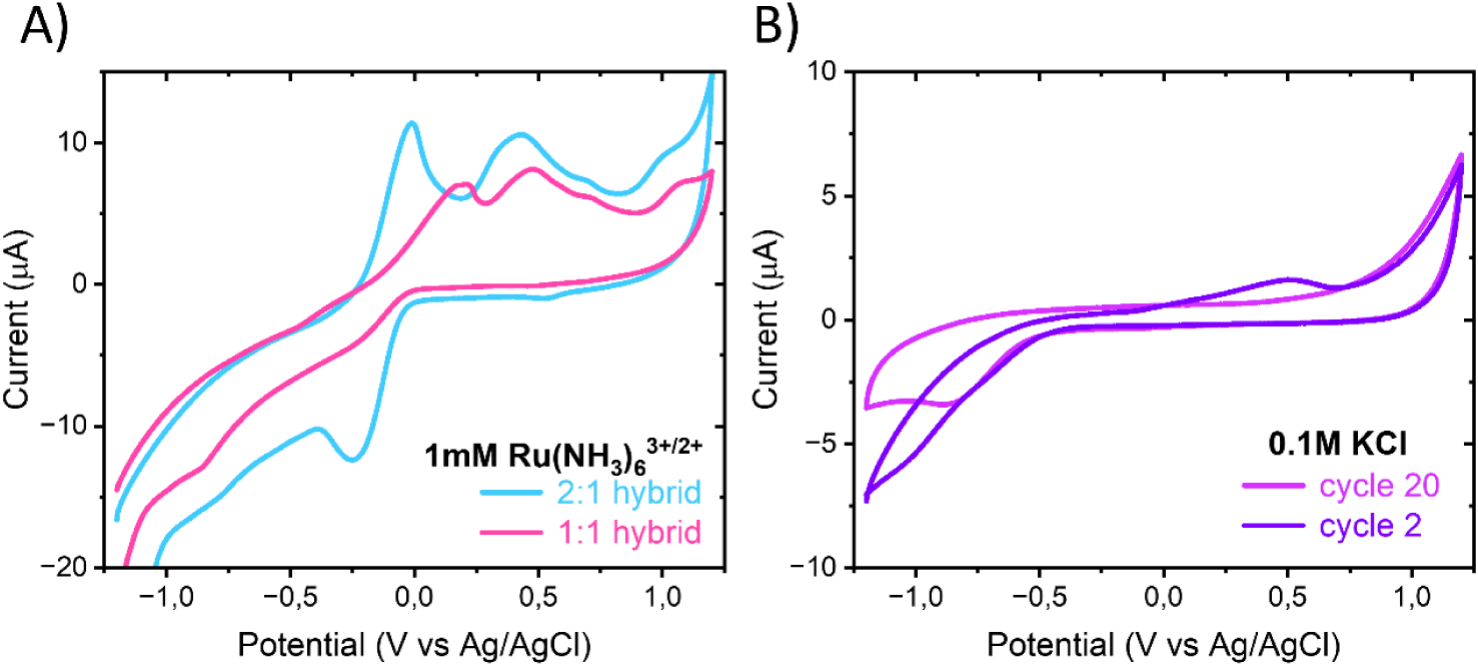
CV measurements in (A) 1 mM Ru­(NH_3_)_6_^3+^ in 0.1 M KCl of 1:1 and 2:1 SWCNT:TOCNF and (B) 0.1 M KCl of 1:1 SWCNT:TOCNF with 100 mV/s. Geometrical area of electrodes 0.07 cm^2^.

**Table 3:** Anodic (Ipa) and Cathodic (Ipc) Peak Current Values Measured with CV with 100 mV/s Instantly in 1 mM Ru­(NH_3_)_6_^3+^ in 0.1 M KCl Solution

	$Ip_{a_{inst}}$ (μA)	$Ip_{c_{inst}}$ (μA)	Δ*E_p_* (mV)
1:1 hybrid	3.2 ± 0.2	3.8 ± 0.0	447 ± 6
2:1 hybrid	10.9 ± 1.0	10.7 ± 1.7	235 ± 4

We attribute these oxidation features to quinone/phenol-type moieties formed on the SWCNT surface during the sonication dispersion step used for hybrid preparation. Consistent with this assignment, we observed an increase in oxygen-containing functionalities when SWCNTs were sonicated alone (Figure S1), and cyclic voltammograms of electrodes containing only SWCNTs also exhibited these additional oxidation peaks (Figure S3). These observations indicate that the formation of redox-active surface groups does not require the presence of TOCNFs. Nevertheless, the presence of TOCNFs in the sonication path is expected to further enhance the formation of oxygen-based functionalities. Such oxygen-containing groups, including phenolic, carbonyl, and quinone-like sites, are well documented to form spontaneously on carbon nanotubes and graphitic carbons after oxidative treatment or prolonged ultrasonication in aqueous media.^24,25^ These surface functionalities are electrochemically active, typically undergoing reversible quinone/hydroquinone redox transitions between 0.3 and 0.6 V in neutral aqueous media,^26,27^ which closely matches the potential range of the additional peaks observed in our system. Thus, the additional peaks observed with Ru­(NH_3_)_6_^3+^ arise from the reduction and subsequent reoxidation of quinone and phenol groups at the surface. During the cathodic sweep to negative potentials, these groups are reduced to hydroquinone, semiquinone, or phenoxide species, which are electron-rich and can transfer electrons to the Ru mediator, generating distinct mediator-assisted peaks. On the anodic sweep, the reduced species are oxidized back to their original forms, producing both direct quinone/phenolic oxidation peaks and the reverse mediator peaks. The main Ru­(NH_3_)_6_^3+^ redox peaks remain visible because the bulk mediator still undergoes its standard one-electron redox reaction independent of surface chemistry. Other types of outer-sphere probes with more positive formal potentials cannot access these reduced states, so no additional peaks are observed, highlighting that the effect depends on both the applied potential and the mediator’s redox properties. An alternative explanation for the additional oxidation peaks could have been residual lignin within the hybrid material; however, this possibility is ruled out by the observation of identical features in SWCNT-only electrodes and by comprehensive physicochemical characterization.^21^

### Neutrally Charged OSR (FcMeOH)

Ferrocene methanol (FcMeOH) was employed as a neutral outer-sphere probe to evaluate the intrinsic electron-transfer characteristics of the hybrid electrodes without the strong electrostatic effects typically associated with charged redox species. Although FcMeOH is commonly treated as electrostatically neutral, its oxidized form (FcMeOH^+^) carries a positive charge, and its reduced form (FcMeOH^0^) can weakly adsorb on hydrophobic or π-conjugated carbon surfaces. Consequently, subtle interfacial and electrostatic effects may still influence its voltammetric response, particularly at low ionic strength or on nanostructured carbon-based materials such as those used in this study.

Cyclic voltammograms of FcMeOH recorded at the hybrid electrodes displayed well-defined and nearly symmetric redox peaks under all conditions (Figure 3). The anodic and cathodic peak currents increased with increasing SWCNT content, reflecting enhanced electronic conductivity and improved percolation pathways in the 2:1 SWCNT:TOCNF hybrid (Table 4). In 0.1 M KCl, the instant measurement yielded anodic peak currents of 10.6 ± 0.5 μA and 12.1 ± 0.8 μA for the 1:1 and 2:1 hybrids, respectively, which further increased after 5 min incubation. The corresponding cathodic peaks followed a similar trend, indicating that incubation promotes gradual wetting of the hybrid network by the electrolyte, leading to an increase in electrochemically active area and thus higher currents. The peak separation decreased markedly with higher CNT content, from 150 mV for the 1:1 hybrid to 86 mV for the 2:1 hybrid in 0.1 M KCl, indicating faster electron-transfer kinetics though not fully reversible behavior. Upon increasing the electrolyte concentration to 1 M KCl, the Δ*E*_*p*_ for the CNT-rich 2:1 hybrid remained low (≈80–87 mV), whereas that for the 1:1 hybrid broadened substantially (210–228 mV). This divergence reflects differences in film morphology and charge transport pathways. At high ionic strength, the electrical double layer becomes thinner, limiting long-range electrostatic access into the film. In the 1:1 hybrid, where the CNT network is poorly percolated and interspersed with cellulose, this compaction hinders ions from reaching internal CNT surfaces, slowing electron and ion transport and broadening Δ*E*_*p*_. In contrast, the 2:1 hybrid features a continuous, percolated CNT network that maintains efficient ion and electron transport even when the double layer is compressed, preserving fast electrochemical kinetics.

**Figure 3:**
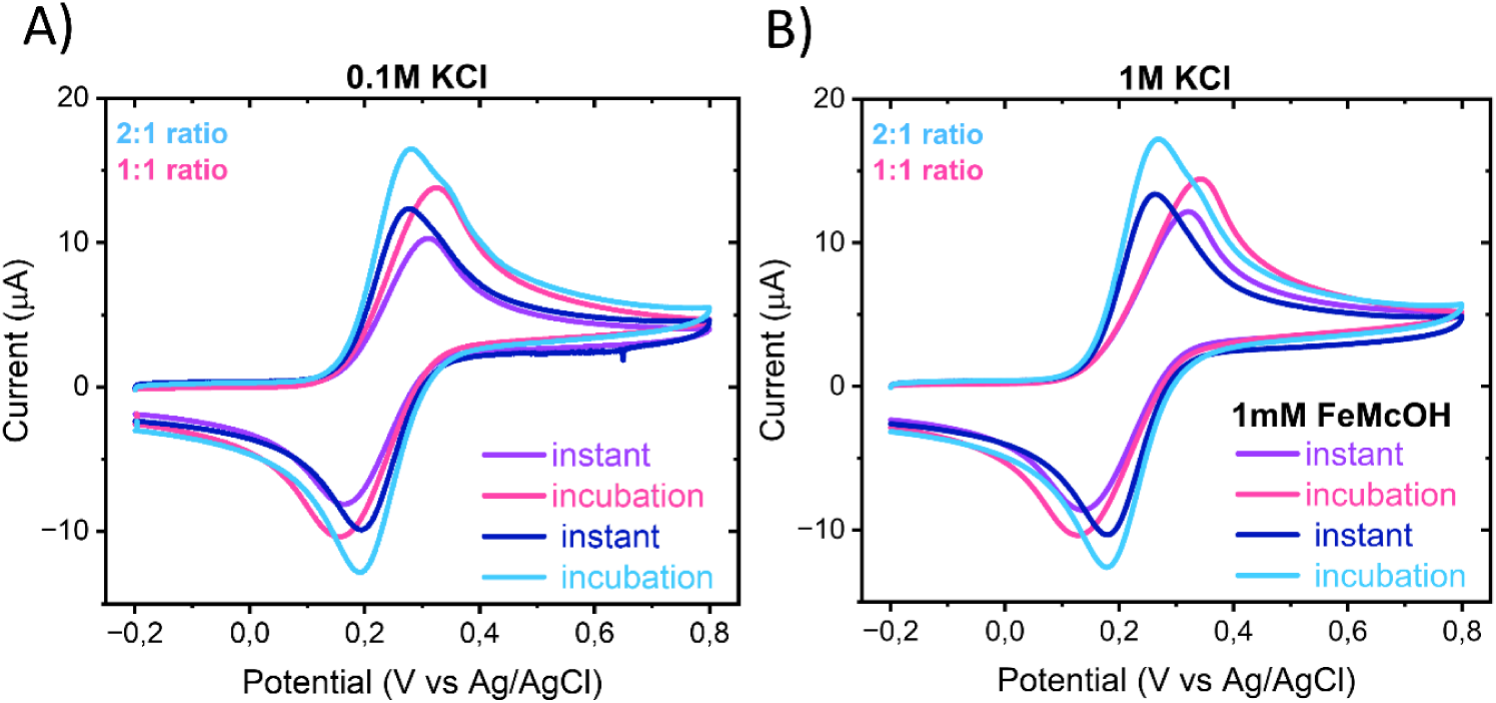
CV measurements instantly or after 5 min of incubation in the analyte solution of 1:1 and 2:1 SWCNT:TOCNF hybrids in 1 mM FeMeOH in (A) 0.1 and (B) 1 M KCl with 100 mV/s. Geometrical area of electrodes 0.07 cm^2^.

**Table 4:** Anodic (Ipa) and Cathodic (Ipc) Peak Current Values Measured with CV with 100 mV/s Instantly and after 5 min Incubation in 1 mM FcMeOH in 0.1 and 1 M KCl Solution

	Electrolyte (M)	$Ip_{a_{inst}}$ (μA)	$Ip_{c_{inst}}$ (μA)	Δ*E_p_* (mV)	$Ip_{a_{incub}}$ (μA)	$Ip_{c_{incub}}$ (μA)	$\Delta E_{p_{incub}}$ (mV)
1:1 hybrid	0.1	10.6 ± 0.5	10.6 ± 0.4	150 ± 3	13.7 ± 0.0	12.8 ± 0.1	168 ± 2
	1	11.0 ± 0.4	10.6 ± 0.4	210 ± 21	13.9 ± 0.2	12.3 ± 0.3	228 ± 9
2:1 hybrid	0.1	12.1 ± 0.8	12.0 ± 0.9	86 ± 2	16.6 ± 0.6	15.2 ± 0.5	93 ± 3
	1	13.0 ± 0.4	12.1 ± 0.3	80 ± 1	17.1 ± 0.3	14.5± 0.2	87 ± 4

### Anionic OSR (IrCl_6_^3–/2–^ and Fe­(CN)_6_^3–/4–^)

Hexachloroiridate (IrCl_6_^3–/2–^) was used to examine how surface charge and ionic strength influence electron transfer with an anionic outer-sphere probe (Figure 4A–B). At the IrCl_6_^3–/2–^ formal potential (≈ +0.75 V vs Ag/AgCl), the SWCNTs remain above their point of zero charge and are therefore positively charged throughout the measurement window. In 0.1 M KCl, the 1:1 and 2:1 SWCNT:TOCNF hybrids exhibited essentially the same peak currents, indicating similar electroactive accessibility to SWCNT domains. However, the CNT-rich 2:1 network showed a smaller Δ*E*_*p*_, consistent with faster electron-transfer kinetics due to more continuous conductive pathways (Table 5). The negatively charged TOCNFs modulate transport by maintaining hydration and counterion structure within the network, helping preserve IrCl_6_^3–/2–^ access despite electrostatic repulsion at TOCNF sites and only moderate attraction to the positively charged SWCNT surface. Increasing the ionic strength to 1 M KCl led to a small decrease in peak currents for both electrodes, reflecting reduced migration and double-layer compression rather than differences in CNT loading. Upon incubation at open-circuit potential (close to the IrCl_6_^3–/2–^ formal potential), peak currents increased slightly, with the 2:1 hybrid benefiting more. This indicates gradual penetration or redistribution of IrCl_6_^3–/2–^ into the denser CNT network over time, likely aided by its higher electronic connectivity and larger effective electroactive area, while the more open 1:1 network is already largely accessible at the initial measurement.

**Figure 4:**
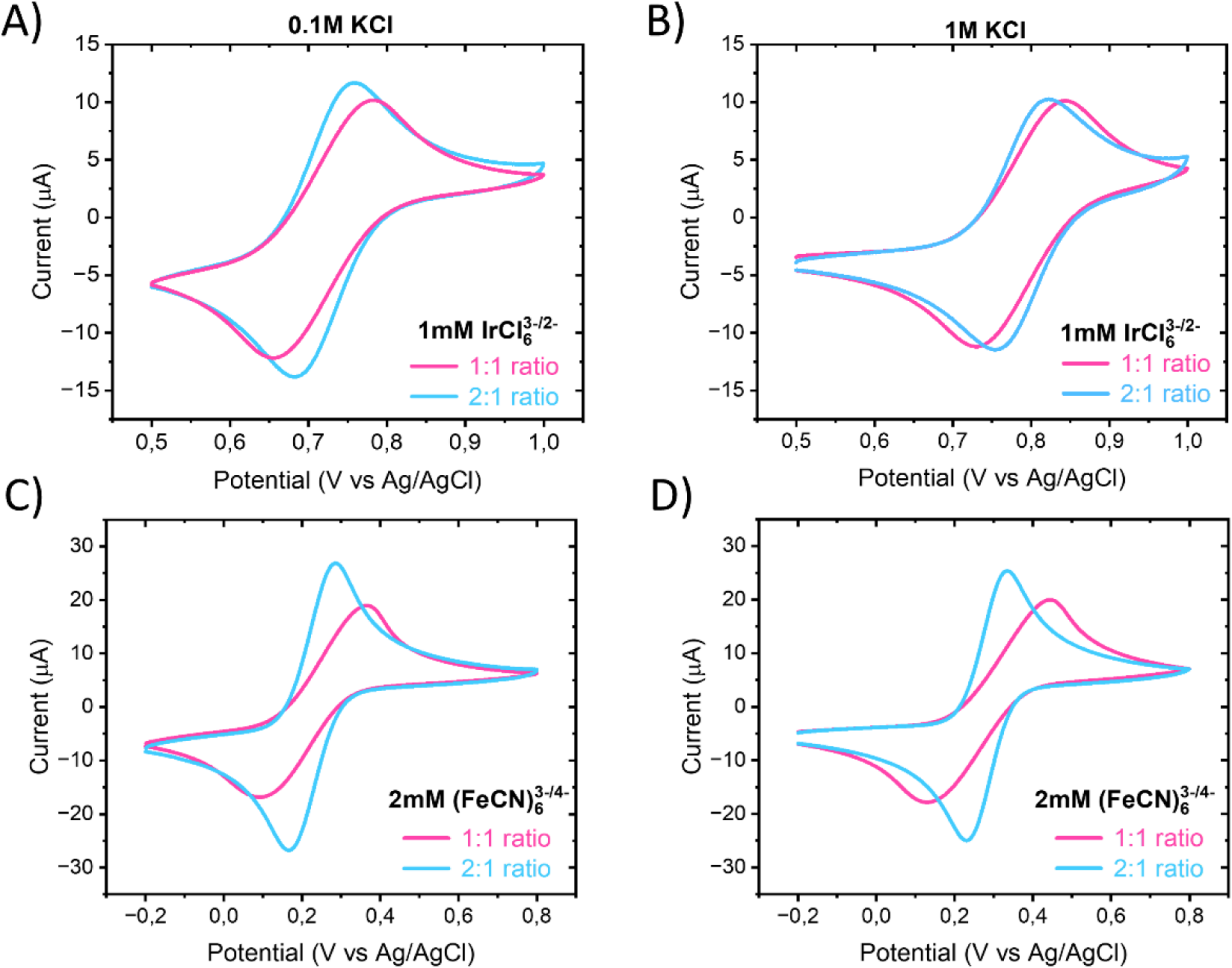
CV measurements after 5 min of incubation in the analyte solution of 1:1 and 2:1 ratio SWCNT:TOCNF hybrids in (A–B) 1 mM IrCl_6_^3–/2–^ and (C–D) 2 mM Fe­(CN)_6_^3–/4–^ in 0.1 and 1 M KCl with 100 mV/s. Geometrical area of electrodes 0.07 cm^2^.

**Table 5:** Anodic (Ipa) and Cathodic (Ipc) Peak Current Values Measured with CV at 100 mV/s Instantly and after 5 min Incubation in 1 mM IrCl_6_ in 0.1 and 1 M KCl Solution

	Electrolyte (M)	$Ip_{a_{inst}}$ (μA)	$Ip_{c_{inst}}$ (μA)	Δ*E_p_* (mV)	$Ip_{a_{incub}}$ (μA)	$Ip_{c_{incub}}$ (μA)	$\Delta E_{p_{incub}}$ (mV)
1:1 hybrid	0.1	11.9 ± 0.1	11.9 ± 0.2	132 ± 5	14.3 ± 0.1	13.8 ± 0.0	147 ± 5
	1	9.8 ± 0.6	8.2 ± 0.6	103 ± 7	12.0 ± 0.3	9.9 ± 0.2	112 ± 5
2:1 hybrid	0.1	12.8 ± 0.4	12.7 ± 0.3	75 ± 2	15.8 ± 0.5	15.6 ± 0.4	75 ± 1.8
	1	9.4 ± 0.9	7.4 ± 0.8	70 ± 2	12.1 ± 0.2	10.3 ± 0.0	67 ± 0.5

The ferri/ferrocyanide redox couple was employed as a complementary anionic probe because, unlike ideal outer-sphere couples, Fe­(CN)_6_^3–/4–^ is well-known to be sensitive to adsorption phenomena and limited electronic coupling at carbon electrodes.^28^ Thus, in addition to probing electrostatic screening, Fe­(CN)_6_^3–/4–^ can reveal surface blocking, heterogeneous kinetics, and film accessibility that may not be apparent from inert OSR probes alone. In 0.1 M KCl, both hybrid electrodes produced well-defined Fe­(CN)_6_^3–/4–^ redox peaks (Figure 4C–D) but with large peak separations (Δ*E*_*p*_ ≈ 258 mV for the 1:1 film and ≈114 mV for the 2:1 film), indicating quasi-reversible to sluggish electron-transfer kinetics. The CNT-rich 2:1 hybrid exhibited higher peak currents compared to the 1:1 film (Table 6). Increasing the ionic strength to 1 M KCl resulted in higher peak currents for both hybrids and a modest reduction in Δ*E*_*p*_, consistent with partial screening of electrostatic repulsion between the negatively charged Fe­(CN)_6_^3–/4–^ ions and the oxygenated CNF domains. At low ionic strength, the more extended double layer surrounding the TOCNFs restricts access of the bulky, hydrated Fe­(CN)_6_^3–/4–^ ions to the conductive CNT network, leading to slower and more heterogeneous charge transfer. In contrast, at higher ionic strength the compact double layer minimizes these barriers, facilitating ion diffusion and more uniform electron transfer throughout the hybrid film. Incubation in the Fe­(CN)_6_^3–/4–^ solution did not significantly change the response at high ionic strength, indicating that ion redistribution within the hybrid network occurs rapidly once electrostatic exclusion is suppressed.

**Table 6:** Anodic (Ipa) and Cathodic (Ipc) Peak Current Values Measured with CV at 100 mV/s Instantly and after 5 min Incubation in 2 mM FeCN_6_ in 0.1 and 1 M KCl Solution

	Electrolyte (M)	$Ip_{a_{inst}}$ (μA)	$Ip_{c_{inst}}$ (μA)	Δ*E_p_* (mV)	$Ip_{a_{incub}}$ (μA)	$Ip_{c_{incub}}$ (μA)	$\Delta E_{p_{incub}}$ (mV)
1:1 hybrid	0.1	18.7 ± 1.1	17.3 ± 0.8	258 ± 15	23.5 ± 1.1	20.6 ± 1.0	288 ± 16
	1	21.8 ± 1.4	20.2 ± 0.7	287 ± 34	21.9 ± 1.3	20.4 ± 0.7	285 ± 36
2:1 hybrid	0.1	23.3 ± 1.1	24.3 ± 0.7	114 ± 3	28.5 ± 1.0	28.9 ± 1.0	124 ± 4
	1	28.1 ± 0.4	27.6 ± 0.4	112 ± 5	28.1 ± 0.4	27.7 ± 0.4	112 ± 5

### Inner Sphere Redox Probe of the Different Ratios

Dopamine was used as an inner-sphere probe to assess the surface reactivity of the hybrid electrodes, as its oxidation involves direct chemical interactions with surface oxygenated or quinone-like groups. In 1X PBS, cyclic voltammograms exhibited a well-defined redox couple centered near 0.2 V, characteristic of the dopamine/dopaminequinone transformation (Figure 5).^29,30^

**Figure 5:**
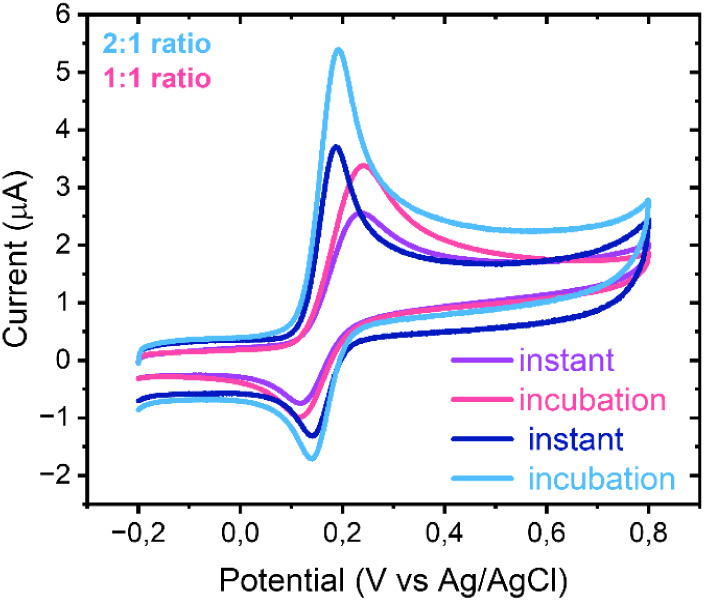
CV measurements instantly or after 5 min of incubation in the analyte solution of 1:1 and 2:1 SWCNT:TOCNF hybrids in 100 μM dopamine in 1X PBS with 100 mV/s. Geometrical area of electrodes 0.07 cm^2^.

The 2:1 hybrid displayed higher anodic currents and a narrower peak separation compared to the 1:1 hybrid (Table 7), indicating more favorable reaction kinetics. The enhanced oxidation current at higher CNT content suggests an increased density of accessible adsorption sites for dopamine, consistent with the larger electroactive area and higher electronic connectivity of the CNT-enriched film. An additional effect of incubation time was observed, as the peak current increased following 5 min exposure to dopamine solution. This behavior may arise from improved wetting of the porous film or the electrostatic attraction between positively charged dopamine and negatively charged surface oxygen groups.

**Table 7:** Anodic (Ipa) and Cathodic (Ipc) Peak Current Values Measured with CV with 100 mV/s Instantly and after 5 min Incubation in 100 μm Dopamine in 1X PBS Solution

	$Ip_{a_{inst}}$ (μA)	$Ip_{c_{inst}}$ (μA)	Δ*E_p_* (mV)	$Ip_{a_{incub}}$ (μA)	$Ip_{c_{incub}}$ (μA)	$\Delta E_{p_{incub}}$ (mV)
1:1 hybrid	2.3 ± 0.1	1.4 ± 0.0	122 ± 2	3.1 ± 0.0	1.6 ± 0.0	126 ± 2
2:1 hybrid	3.7 ± 0.3	1. 8 ± 0.1	46 ± 2	5.1 ± 0.1	2.3 ± 0.0	51 ± 3

Overall, dopamine oxidation at the hybrid electrodes is governed by both electronic and chemical factors: electron-transfer conductivity provided by the CNT framework and the density of oxygen functionalities capable of transient interactions with the analyte. The smaller Δ*E*_*p*_ and higher currents in the 2:1 hybrid reflect this synergistic balance, while the modest cathodic response (∼1 μA, Table 7) is consistent with the quasi-irreversible nature of dopamine redox cycling in neutral media.

## Conclusions

Self-standing SWCNT:TOCNF hybrid films were demonstrated as sustainable, bioderived electrodes that integrate the high conductivity of carbon nanotube networks with the hydrophilic, carboxylated scaffold of cellulose nanofibers. Structural and electrical analyses confirmed a physically interconnected architecture in which TOCNFs disperse and stabilize conductive CNT domains without compromising their intrinsic electronic properties. Systematic electrochemical characterization using outer- and inner-sphere redox probes revealed that electron transfer is primarily governed by CNT network percolation and ionic accessibility, while the surface chemistry of the TOCNF matrix modulates adsorption and interfacial kinetics. Composition-dependent behavior was observed: CNT-rich hybrids exhibited faster electron transfer and higher conductivity, whereas more balanced formulations enhanced accessibility and adsorption-driven interactions, highlighting the trade-off between electronic transport and analyte interaction.

Unlike conventional sensors that rely on external conductive substrates or binders, these self-standing hybrids serve simultaneously as the electrical and sensing interface, providing a mechanically robust, tunable, and fully bioderived platform. Collectively, these results establish a clear structure–function relationship linking hybrid composition, interfacial charge, and electrochemical response, offering mechanistic insight into charge-mediated detection in carbon–cellulose interfaces. The modular and sustainable nature of SWCNT:TOCNF hybrids underscores their potential as adaptable electrochemical sensing platforms.

## Supplementary Material



## Notes

Additional characterization of SWCNTs with FTIR and additional CV measurements in the OSR probe are provided (PDF)
