# Supplementary material for: Charge-Regulated Electrochemistry in Self-Standing Carbon Nanotube–Nanocellulose Hybrid Electrodes
Source: Langmuir. 2026 May 12;42(20):14491–9. doi: 10.1021/acs.langmuir.6c01676 (PMC13217609; doi:10.1021/acs.langmuir.6c01676)
Supplement: Supplementary file 1 [file la6c01676_si_001.pdf]

Supplementary Information for: "Charge-Regulated Electrochemistry in Self-Standing Carbon Nanotube–Nanocellulose Hybrid Electrodes"

Laura Ferrer Pascual[a], Golnoosh Akhlamadi[a], Khadijeh Nekoueian[a], Maedeh Akhoundian[a], Mengqi Sun[a], Kristoffer Meinander[b], Henrikki Liimatainen[c] & Tomi Laurila[a,d,*]

[a]*Department of Electrical Engineering and Automation, Aalto University, Maarintie 8, Espoo, 02150, Finland*

[b]*Department of Bioproducts and Biosystems, School of Chemical Engineering, Aalto University, PO Box 16300, 00076 Aalto, Finland*

[c]*Fiber and Particle Engineering, University of Oulu, P.O. Box 4300, 90014, Oulu, Finland*

[d]*Department of Chemistry and Materials Science, Aalto University, Kemistintie 1, Espoo, 02150, Finland*

**corresponding author email: tomi.laurila@aalto.fi*

TABLE OF CONTENTS

Structural Characterization S**2**

Figure S1 .... S2

Electrochemical Characterization **S3**

Figure S2 .... S3

Figure S3 .... S3

Structural Characterization

Fourier Transform Infrared Spectroscopy (FTIR)

We performed FTIR on the as-purchased SWCNT powder and on dispersions of SWCNTs sonicated for 30 or 60 minutes and dried in a vacuum oven at 50 °C for 10 h. The pristine SWCNTs showed a weak peak around 1700 cm^{-1}, which could be associated with amorphous carbon residues that are easily functionalized by oxygen. The intensity of this peak increased in the 30- and 60-minute sonicated samples, indicating the formation of oxygen-containing functionalities due to the sonication of the SWCNTs.

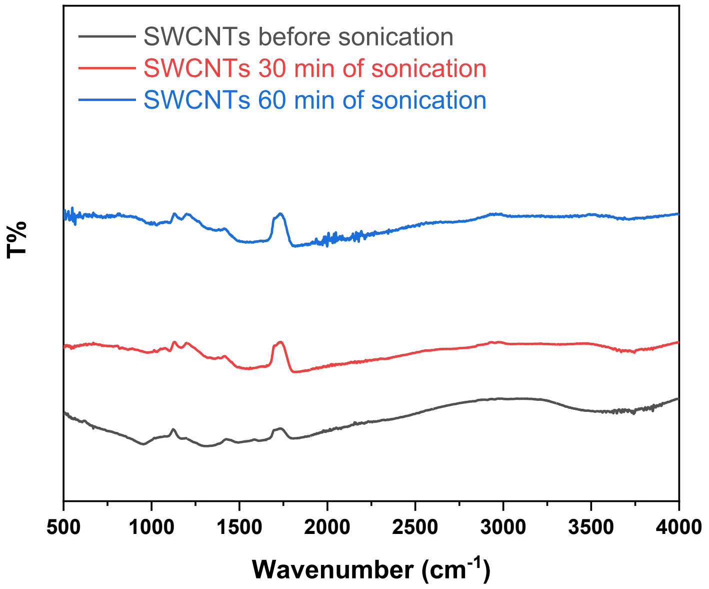

Figure S1. FTIR of SWCNTs before and after 30- or 60-minutes sonication time.

Electrochemical Characterization

Cationic probe $Ru(NH_3)_6^{3+/2+}$

No additional oxidation peaks are observed in 1mM $Ru(NH_3)_6^{3+/2+}$ when using a narrower potential window (-0.5 to 0.3V vs Ag/AgCl), confirming that their appearance is triggered by prior reductive activation of the electrode surface.

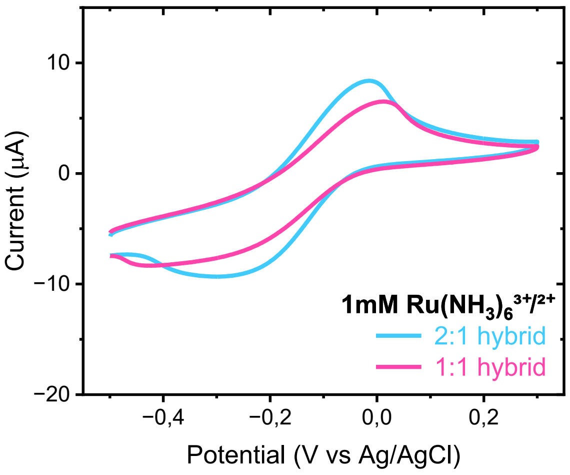

Figure S1. CV measurements in 1mM $Ru(NH_3)_6^{3+}$ in 0.1M KCl of 1:1 and 2:1 SWCNT-TOCNF with 100mV/s.

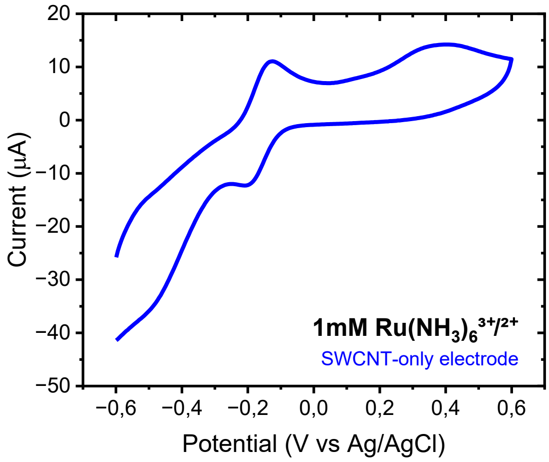

Figure S2. CV measurements in 1mM $Ru(NH_3)_6^{3+}$ in 1M KCl of SWCNT-only electrode with 100mV/s.
